# Supplementary material for: Charting the global footprint of borderline oxacillin-resistant Staphylococcus aureus (BORSA): the first systematic review and meta-analysis
Source: PeerJ. 2024 Dec 16;12:e18604. doi: 10.7717/peerj.18604 (PMC11657201; doi:10.7717/peerj.18604)
Supplement: Supplemental Information 1 [file peerj-12-18604-s001.docx]

**Keywords**

“**borderline oxacillin-resistant Staphylococcus aureus** OR **oxacillin-resistant** OR BORSA”

**Search strategy**

| **No** | **Databases** | **Search strategy** | **Total (*N*)** |
| --- | --- | --- | --- |
| 1 | **PubMed** | **(((borderline oxacillin-resistant Staphylococcus aureus[Title/Abstract])) OR (BORSA[Title/Abstract])) OR (oxacillin-resistant[Title/Abstract])** | **791** |
| 2 | **Scopus** | TITLE-ABS ( borderline AND oxacillin-resistant AND staphylococcus AND aureus ) OR TITLE-ABS (BORSA) OR TITLE-ABS (oxacillin-resistant) | **1,496** |
| 3 | **ScienceDirect** | (Borderline oxacillin-resistant Staphylococcus aureus OR oxacillin-resistant OR BORSA) | **1,100** |
| 4 | **Google Scholar** | allintitle: (borderline oxacillin-resistant) | **46** |
| 5 | **Web of Science** | ((TI=(borderline oxacillin-resistant Staphylococcus aureus )) OR TI=(oxacillin-resistant)) OR TI=(BORSA) | **332** |

Total: 3765
