# Supplementary material for: Charting the global footprint of borderline oxacillin-resistant Staphylococcus aureus (BORSA): the first systematic review and meta-analysis
Source: PeerJ. 2024 Dec 16;12:e18604. doi: 10.7717/peerj.18604 (PMC11657201; doi:10.7717/peerj.18604)
Supplement: Supplemental Information 2 [file peerj-12-18604-s002.docx]

**Table S2: Quality of included studies by JBI critical appraisal checklist for studies reporting prevalence data**

| **Study ID** | | **Checklist*** | | | | | | | | | **Overall** |
| --- | --- | --- | --- | --- | --- | --- | --- | --- | --- | --- | --- |
|  |  | **1** | **2** | **3** | **4** | **5** | **6** | **7** | **8** | **9** |  |
| 1 | Al-Safaar (2013) | Y | Y | Y | Y | Y | Y | Y | U | Y | 88.9% |
| 2 | Argudín (2018) | Y | Y | Y | Y | Y | Y | Y | NA | Y | 88.9% |
| 3 | Balslev (2005) | Y | Y | Y | Y | Y | Y | Y | Y | Y | 100% |
| 4 | Buchan (2010) | Y | Y | Y | Y | Y | Y | Y | Y | Y | 100% |
| 5 | Bystroń (2010) | Y | Y | Y | Y | Y | Y | Y | U | Y | 88.9% |
| 6 | Dicko (2023) | Y | Y | Y | Y | Y | Y | Y | Y | Y | 100% |
| 7 | Dillard (1996) | Y | Y | Y | Y | Y | Y | Y | Y | Y | 100% |
| 8 | Huang (2000) | Y | Y | Y | Y | Y | Y | Y | NA | Y | 88.9% |
| 9 | Huang_a (2018) | Y | Y | Y | Y | Y | Y | Y | Y | Y | 100% |
| 10 | Khorvash (2008) | Y | Y | Y | Y | Y | Y | Y | Y | Y | 100% |
| 11 | Konstantinovski (2021) | Y | Y | Y | Y | Y | Y | Y | NA | Y | 88.9% |
| 12 | Konstantinovski_a (2021) | Y | Y | Y | NA | Y | Y | Y | Y | Y | 88.9% |
| 13 | Krupa (2014) | Y | Y | Y | Y | Y | Y | Y | NA | Y | 88.9% |
| 14 | Krupa_a (2015) | Y | Y | Y | Y | Y | Y | Y | NA | Y | 88.9% |
| 15 | Leahy (2011) | Y | Y | Y | Y | Y | Y | Y | Y | Y | 100% |
| 16 | Liu (1990) | Y | Y | Y | Y | Y | Y | Y | U | Y | 88.9% |
| 17 | Ljiljana (2008) | Y | Y | Y | Y | Y | Y | Y | U | Y | 88.9% |
| 18 | Maalej (2012) | Y | Y | Y | NA | Y | Y | Y | NA | Y | 77.8% |
| 19 | Martineau (2000) | Y | Y | Y | Y | Y | Y | Y | U | Y | 88.9% |
| 20 | Nakamura (2002) | Y | Y | Y | Y | Y | Y | Y | Y | Y | 100% |
| 21 | Perillo (2012) | Y | Y | Y | Y | Y | Y | Y | Y | Y | 100% |
| 22 | Sá-Leão (2001) | Y | Y | Y | Y | Y | Y | Y | Y | Y | 100% |
| 23 | Santhosh (2008) | Y | Y | Y | Y | Y | Y | Y | Y | Y | 100% |
| 24 | Santos (2021) | Y | Y | Y | Y | Y | Y | Y | U | Y | 88.9% |
| 25 | Sawhney (2022) | Y | Y | Y | Y | Y | Y | Y | Y | Y | 100% |
| 26 | Sieber (2011) | Y | Y | Y | Y | Y | Y | Y | U | Y | 88.9% |
| 27 | Stańkowska (2019) | Y | Y | Y | Y | Y | Y | Y | Y | Y | 100% |
| 28 | Tawil (2013) | Y | Y | Y | Y | Y | Y | Y | Y | Y | 100% |
| 29 | Zehra (2020) | Y | Y | Y | Y | Y | Y | Y | Y | Y | 100% |

* **1.** Appropriate sampling frame to address target population, **2.** Appropriate sampling way of study participants, **3.** Adequate sample size, **4.** Detail description of study participants and settings, **5.** Data analysis with sufficient coverage of identified sample, **6.** Use of valid methods to identify the condition, **7.** Standard, reliable way of measurement of condition for all participants, **8.** Availability of appropriate statistical analysis, **9.** Adequate response rate and management of low response rate
